# Warming and Salt Intrusion Affect Microcystin Production in Tropical Bloom-Forming *Microcystis*

**DOI:** 10.3390/toxins14030214

**Published:** 2022-03-16

**Authors:** Bui Trung, Marlies E. Vollebregt, Miquel Lürling

**Affiliations:** Aquatic Ecology & Water Quality Management Group, Department of Environmental Sciences, Wageningen University, P.O. Box 47, 6700 AA Wageningen, The Netherlands; marlies.vollebregt@wur.nl (M.E.V.); miquel.lurling@wur.nl (M.L.)

**Keywords:** climate change, cyanobacteria, cyanotoxins, Mekong Delta, salt intrusion

## Abstract

The Vietnamese Mekong Delta is predicted to be one of the regions most impacted by climate change, causing increased temperature and salinity in inland waters. We hypothesized that the increase in temperature and salinity may impact the microcystin (MC) production of two *Microcystis* strains isolated in this region from a freshwater pond (strain MBC) and a brackish water pond (strain MTV). The *Microcystis* strains were grown at low (27 °C), medium (31 °C), high (35 °C) and extremely high (37 °C) temperature in flat photobioreactors (Algaemist). At each temperature, when cultures reached a stable state, sea salt was added to increase salinity to 4‰, 8‰, 12‰ and 16‰. MC concentrations and cell quota were reduced at high and extremely high temperatures. Salinity, in general, had comparable effects on MC concentrations and quota. At a salinity of 4‰ and 8‰, concentrations of MC per mL of culture and MC cell quota (based on chlorophyll, dry-weight and particle counts) were higher than at 0.5‰, while at the highest salinities (12‰ and 16‰) these were strongly reduced. Strain MBC produced five MC variants of which MC-RR and MC-LR were most abundant, followed by MC-YR and relatively low amounts of demethylated variants dmMC-RR and dmMC-LR. In strain MTV, MC-RR was most abundant, with traces of MC-YR and dmMC-RR only in cultures grown at 16‰ salinity. Overall, higher temperature led to lower MC concentrations and cell quota, low salinity seemed to promote MC production and high salinity reduced MC production. Hence, increased temperature and higher salinity could lead to less toxic *Microcystis*, but since these conditions might favour *Microcystis* over other competitors, the overall biomass gain could offset a lower toxicity.

## 1. Introduction

Blooms of harmful cyanobacteria are a problem in lakes, rivers and estuaries worldwide [1]. They can cause a multitude of water quality concerns, such as oxygen deficiency leading to fish kills, while production of toxins may pose a hazard to aquatic organisms, wildlife, pets and humans [2]. Cyanobacterial blooms are increasing in occurrence, size and duration [3], due to ongoing eutrophication and climate change [4]. Furthermore, water bodies in the Mekong Delta in Southern Vietnam suffer from cyanobacterial blooms, and are mostly dominated by members of the genus *Microcystis* [5], which is one of the most frequently encountered bloom-forming cyanobacteria worldwide [6]. Strains of *Microcystis* may produce hepatoxic microcystins (MCs) [6].

The Vietnamese Mekong Delta is predicted to be strongly impacted by climate change [7,8,9,10]. The annual average temperature in Vietnam has increased by 0.5–0.7 °C nationwide in the last 50 years [11]. Warming may promote cyanobacterial dominance [1,12] and at temperatures above 20 °C, warming may lead to rapid rise of cyanobacteria biomass [13]. For instance, summer bloom biomass of *Microcystis* was more than 700% higher in mesocosms that were 4 degrees warmer than mesocosms at ambient temperature [14]. Warming may also affect MC cell quota; under warming conditions, total MC quota and toxicity of *Microcystis* are generally reduced [15,16,17,18]. Likewise, in *Microcystis* strains isolated from water bodies in southern Vietnam, raising temperatures above 27 °C led to increased growth rates, but decreased total MC production and MC cell quota [19].

Another consequence of climate change is sea level rise, where satellite imaging has indicated an average sea level rise of 2.9 mm year^−1^ for the whole coastal zone in Southern Vietnam [11]. Such sea level rise can enhance saline intrusion into the freshwater ecosystems of the Mekong Delta. Seawater intrusion can reach 40–50 km upstream from the mouth of Tien and Hau rivers (Mekong river branches) along the delta, resulting in raising salinity of water up to ≥4‰, and the intrusion is projected to increase in near future [20,21,22,23]. In general, increased salinity may restrict growth and proliferation of cyanobacteria as most freshwater cyanobacteria are sensitive to salt stress [4,24]. Some *Microcystis* strains, however, show a considerable ability to resist rising salinity and have the capacity to thrive under elevated salinity [25,26]. Hence, *Microcystis* is found regularly in brackish water bodies [6,27,28,29], including in ponds in the Mekong Delta with a salinity of >7‰ [5]. As with temperature, salinity may not only influence *Microcystis* growth, but also MC production. *Microcystis* growth, MC cell quota and MC production can either remain unaffected until salinities of 10 g L^−1^ [25,30], or *Microcystis* growth and MC quota may be reduced with increased salinity [26,30]. 

Although effects of temperature and salinity on *Microcystis* have been studied separately, relatively little is known of their combined effects on MC production in tropical *Microcystis*. Inasmuch as higher salt intrusion in the Mekong region is expected in the warm, dry period, *Microcystis* strains isolated from water bodies in this area were exposed to these two factors simultaneously in highly controlled photo-bioreactors (see Section 5.2). This set-up allowed testing the hypothesis that increases in temperature would reduce MC quota, where salinity would not have an effect until elevated to such concentration that it causes death of the cells. 

## 2. Results

### 2.1. Effect of Temperature and Salinity on Biomass Indicators

**Figure 1:**
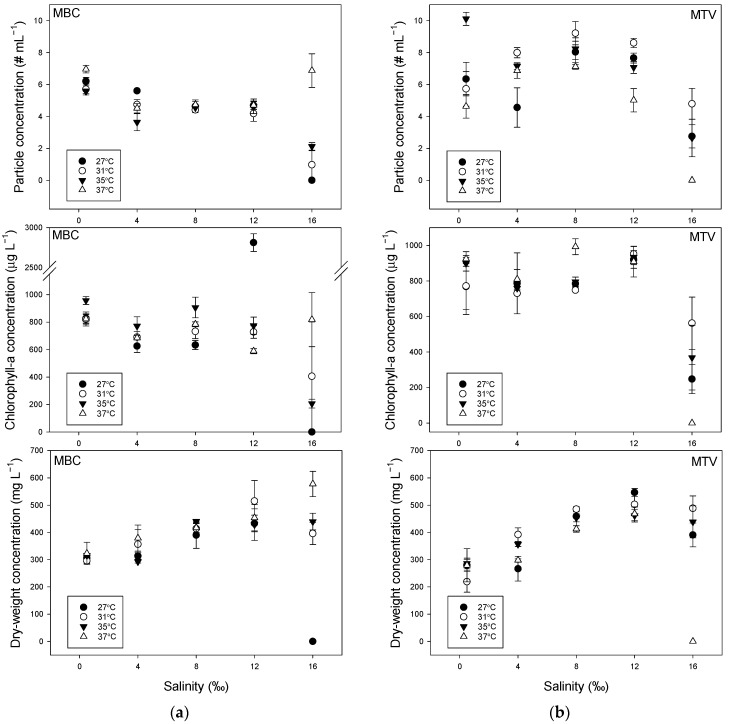
(**a**) Steady state particle concentrations (upper panel), chlorophyll-*a* concentrations (middle panel) and dry-weight concentrations (lower panel) of *Microcystis aeruginosa* strain MBC grown in photobioreactors in media of different salinities (0.5–16‰) at four temperatures (27–37 °C). Values are means of three consecutive days. Error bars indicate 1SD; (**b**) Steady state particle concentrations (upper panel), chlorophyll-*a* concentrations (middle panel) and dry-weight concentrations (lower panel) of *Microcystis aeruginosa* strain MTV grown in photobioreactors in media of different salinities (0.5–16‰) at four temperatures (27–37 °C). Values are means of three consecutive days. Error bars indicate 1SD.

Both strains were able to build dense populations in salinities of 0.5‰ to 12‰ irrespective of temperature (Figure 1). Dry-weight concentrations increased with salinity in the range 0.5‰ to 12‰ in both strains (Figure 1; Appendix A, Table A1) and this was not influenced by temperature as the slopes were similar (Table A2). Furthermore, for the other two biomass indicators, i.e., number of particles mL^−1^ and chlorophyll-*a* concentration, Parallel Lines Analyses revealed that there was no difference in slopes and intercept at each of the four temperatures tested for those indicators against salinity in the range 0.5‰ to 12‰ (Table A2). Hence, each strain seemed to respond similarly to salinity in the range 0.5‰ to 12‰, where temperature had a marginal effect on biomass (Figure 1). However, at 16‰, clear differences occurred (Figure 1). In strain MBC, there was a wash-out at 27 °C, strongly reduced particle and chlorophyll-*a* concentrations at 31 °C and 35 °C, while the culture at 37 °C had higher biomass indicators than cultures at other temperatures at this salinity and even among the highest biomass of all salinity–temperature combinations tested (Figure 1a). In strain MTV, at 16‰, there was a wash-out at 37 °C, while reduced particle, chlorophyll-*a* and dry-weight concentrations were observed at 27 °C, 31 °C and 35 °C (Figure 1b).

### 2.2. Microcystin Concentrations

**Figure 2:**
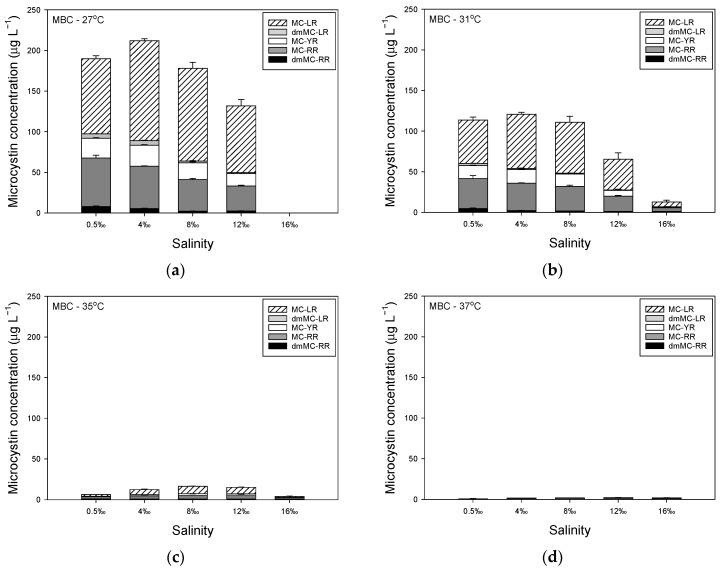
(**a**) Steady state concentrations of different microcystin variants (µg L^−1^) in *Microcystis aeruginosa* strain MBC grown at different salinities (0.5–16‰) at 27 °C. Values are means of three consecutive days. Error bars indicate 1SD; (**b**) Concentrations of different microcystin variants in strain MBC grown at different salinities (0.5–16‰) at 31 °C; (**c**) Concentrations of different microcystin variants in strain MBC grown at different salinities (0.5–16‰) at 35 °C; (**d**) Concentrations of different microcystin variants in strain MBC grown at different salinities (0.5–16‰) at 37 °C.

MCconcentrations in strain MBC were strongly affected by temperature, although higher salinities also led to lower MC concentrations (Figure 2). At 27 °C, MC concentration first increased from on average 190 µg L^−1^ in standard medium of 0.5‰ to 212 µg L^−1^ at 4‰, whereafter MC concentrations declined to 178 µg L^−1^ at 8‰ and to 132 µg L^−1^ at 12‰, while it was virtually zero at 16‰ due to the wash out (Figure 2a). Five MC variants were found in strain MBC, with MC-RR and MC-LR being most abundant (Figure 2a). The share of dmMC-RR, MC-RR, MC-YR and dmMC-LR declined with salinity, while that of MC-LR increased (Table A3). 

When grown at 31 °C, MC concentration first increased slightly from on average 114 µg L^−1^ in standard medium of 0.5‰ to 121 µg L^−1^ at 4‰, whereafter MC concentrations declined to 111 µg L^−1^ at 8‰, to 65 µg L^−1^ at 12‰ and to 13 µg L^−1^ at 16‰ (Figure 2b). The same five MC variants were detected and as in the culture grown at 27 °C, also at 31 °C the variants MC-RR and MC-LR were most abundant (Figure 2a, Appendix B, Table A3). Elevated salinity caused a lower share of dmMC-RR, MC-RR and dmMC-LR in the total MCpool and a seemingly unaltered share of MC-YR, while the share of MC-LR increased (Table A3).

In a high temperature of 35 °C, MC concentrations were much lower than at colder temperatures: on average, 6 µg MC L^−1^ in standard medium of 0.5‰, 12 µg L^−1^ at 4‰, 17 µg L^−1^ at 8‰, 15 µg L^−1^ at 12‰, and 4 µg L^−1^ at 16‰ (Figure 2c). There was a higher share of dmMC-RR to the overall MC pool than at colder temperatures and MC-RR also seemed slightly more present, while MC-LR had a somewhat lower share, and dmMC-LR was reduced to levels below the level of quantification (Table A3). At 37 °C, MC concentrations were even further reduced and were on average 0.8 µg MC L^−1^ in standard medium of 0.5‰, 1.8 µg L^−1^ at 4‰, 2.0 µg L^−1^ at 8‰, 2.4 µg L^−1^ at 12‰ and 1.9 µg L^−1^ at 16‰ (Figure 2d). The demethylated variants dmMC-RR and dmMC-LR were both reduced to concentrations below the level of quantification, which was also the case for MC-RR in standard medium of 0.5‰ (Table A3). In medium with higher salinities, however, MC-RR was the most abundant MC variant, followed by MC-LR (Table A3).

**Figure 3:**
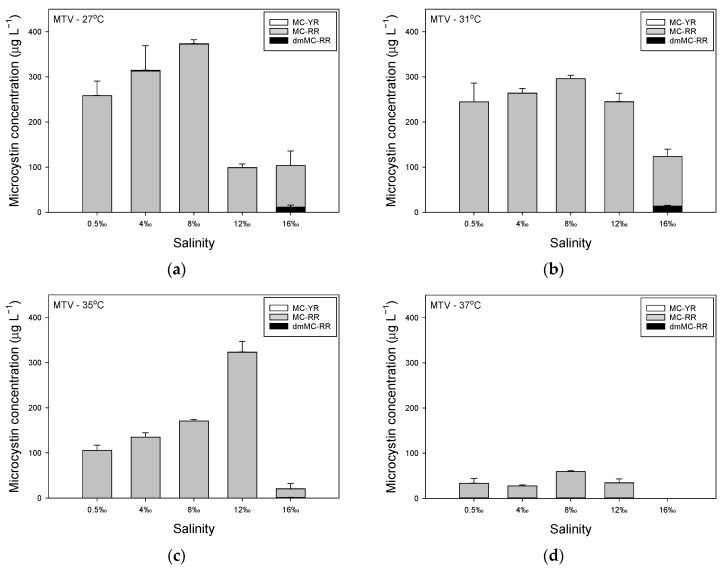
(**a**) Steady state concentrations of different microcystin variants (µg L^−1^) in *Microcystis aeruginosa* strain MTV grown at different salinities (0.5–16‰) at 27 °C. Values are means of three consecutive days. Error bars indicate 1SD; (**b**) Concentrations of different microcystin variants in strain MTV grown at different salinities (0.5–16‰) at 31 °C; (**c**) Concentrations of different microcystin variants in strain MTV grown at different salinities (0.5–16‰) at 35 °C; (**d**) Concentrations of different microcystin variants in strain MTV grown at different salinities (0.5–16‰) at 37 °C.

In strain MTV, MCconcentrations increased with salinity rise from 0.5‰ to 8‰ when this strain was grown at 27 °C (Figure 3a). However, in medium of 12‰ and 16‰, MC concentrations dropped to values of only 25% of those in medium of 8‰ (Figure 3a). When strain MTV was grown at 31 °C, there was first an increase in MC concentration over 0.5‰ to 8‰, followed by a decrease at 12‰ and 16‰ (Figure 3b). At 35 °C, MC concentrations increased with salinity from 0.5‰ to 12‰ and only dropped sharply at 16‰ (Figure 3c). When strain MTV was cultured at 37 °C, MC concentrations were much lower and absent at 16‰ (Figure 3d). Strain MTV produced mostly the variant MC-RR, which comprised more than 99% of the total MCpool in medium of 0.5‰ to 12‰ at 27 °C, 31 °C and 35 °C. However, in medium of 16‰ dmMC-RR was also found making up 10.8%, 10.9% and 6.7% of the total MCpool, while MC-RR made up 89‰ to 93% of the total MCpool. In all samples, low amounts of MC-YR were found, <1% of the MCpool (Figure 3).

### 2.3. Microcystin Quota

**Figure 4:**
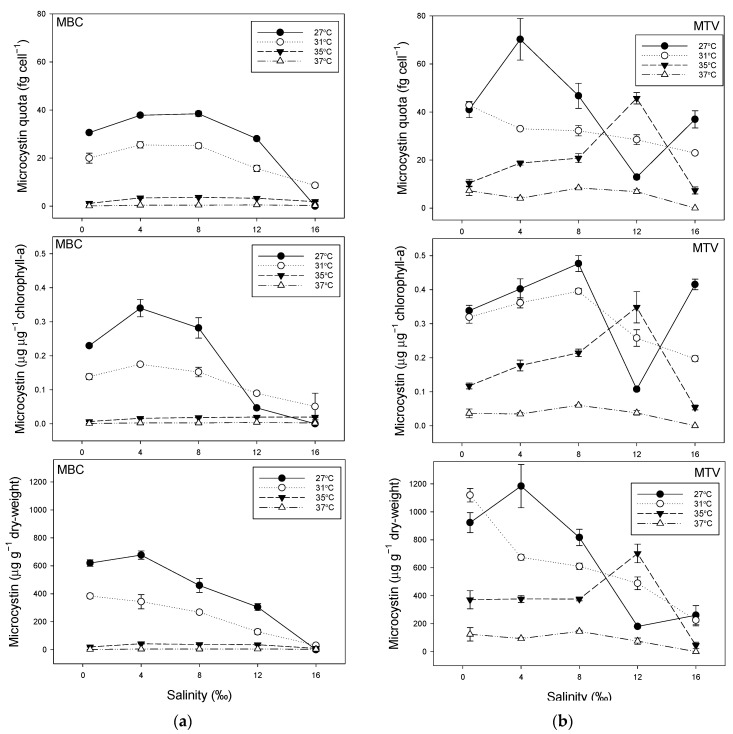
(**a**) Steady state microcystin (MC) cell quota (upper panel), MC per unit chlorophyll-*a* (middle panel) and the MC mass per dry-weight (lower panel) of *Microcystis aeruginosa* strain MBC grown in photobioreactors in media of different salinities (0.5–16‰) at four temperatures (27–37 °C). Values are means of three consecutive days. Error bars indicate 1SD; (**b**) Steady state MC cell quota (upper panel), MC per unit chlorophyll-*a* (middle panel) and the MC mass per dry-weight (lower panel) of *M. aeruginosa* strain MTV grown in photobioreactors in media of different salinities (0.516‰) at four temperatures (27–37 °C). Values are means of three consecutive days. Error bars indicate 1SD.

MC expressed per biomass indicators—particle concentration, chlorophyll-*a* concentration and dry-weight concentration—showed lower quota in both strains when these were grown at warmer temperatures (Figure 4). In strain MBC, MC quota were particularly low in cultures grown at 35 °C and 37 °C (Figure 4a), while in strain MTV this was observed only at 37 °C (Figure 4b). Where in strain MBC the MC quota waslowest in cultures grown at higher salinities (Figure 4a), this pattern was less clear in strain MTV, which showed a more erratic pattern, but based on dry-weight also in this strain MC quota were lowest at the highest salinity of 16‰ (Figure 4b).

## 3. Discussion

The results of this study revealed that both *Microcystis* strains could grow and maintain high biomass at temperatures up to 37 °C. This was the highest temperature tested in our experiment, and in line with other findings on tolerance of *Microcystis* to relative high temperatures. For instance, Mowe et al. (2015) [17] found good growth in four out of five *Microcystis* strains at 36 °C, including *M. aeruginosa*. Likewise, van der Westerhuizen and Eleoff (1985) [16] reported good growth of *M. aeruginosa* at 36 °C. It is likely that our *M. aeruginosa* had even higher temperature tolerance than 37 °C as some studies have reported good growth of *M. aeruginosa* at 40 °C [31,32], and one study even reported net photosynthesis of *M. aeruginosa* and *M. wesenbergii* at 45 °C [33]. The observed temperature tolerance is in line with our previous findings in batch systems that included the same strains (MBC is MBC2 in Bui et al., 2018a) [19]. The highest temperature tested, 37 °C, may seem rather high for aquatic systems, but in the southern region of Vietnam heat waves during spring/summer may push water temperature in small water bodies, such as fishponds, above 37 °C [19]. Over the last years, such heat waves have become a regular phenomenon in the Indochina peninsula [34], which is in line with the prediction that the Mekong Delta will be one of the regions that will be most extremely influenced by climate change [7,8]. The dry season rainfall in the Mekong River Basin is also projected to decrease [35], and last year’s severe drought and historic low discharge led to “saltwater intrusion appearing 10–20 days earlier than the historic 2015–2016 lows and 2.5–3.5 months earlier than the annual average” [36].

The 2020 drought and saltwater intrusion had severe impacts on animal husbandry, aquaculture, drinking water availability, fishing and fruit and vegetable farming in the Mekong Delta [36]. Although in 2020 no surveys on cyanobacteria blooms were undertaken, in our 2016 survey, we found *Microcystis* dominating in sites in the Mekong River, a canal and fishponds in the Delta at salinities of 6.7–7.6‰ [5]. The results of the current study underpin that *M. aeruginosa* strains from the Mekong Delta can tolerate salinities up to at least 12‰ and, depending on strain and temperature, even 16‰. The highest salinity tested (16‰) yielded only two distinct temperatures at which a strain could not maintain its biomass and was washed out of the reactor: In strain MTV, the wash-out occurred at 37 °C, while for strain MBC it occurred at 27 °C. This salinity tolerance is at the upper range of what has been reported for *M. aeruginosa*, whichvaries from low salinities of around 2‰ to values above 16‰ [29]. 

Different responses of *M. aeruginosa* to salinity have been reported. Reduced growth when salinity was increased from 0.6 to 12.6 g L^−1^ has been observed, but cells remained intact, while at 20.6 g L^−1^ they died [26]. *M. aeruginosa* strains PCC7820 and PCC7806 expressed positive growth in salinity ranges of 0.6–7.6 g L^−1^ and 0.616.9 g L^−1^, respectively, where growth in PCC7820 declined with increasing salinity, while in PCC7806 growth remained constant until salinity of 8.4 g L^−1^ [30]. Positive growth was observed in *M. aeruginosa* in salinity of 16.6‰, althoughwith around 0.4 doublings per day, the growth was lower than the approximately 1 doubling per day at 11‰ [37], and likewise in salinity up to 18‰ [38]. The latter experiments were conducted with *M. aeruginosa* harvested from the field, which were acclimatized for three generations [37], or 10 days [38]. Most likely, those *M. aeruginosa* still had their typical colonial morphology, as normally observed in the field; colonies are more protected against external chemical stressors than unicells, because they are embedded in a protective mucilage layer [39]. Large colonies, originating from a freshwater bloom, may survive for days in a marine environment [40], potentially causing harm to marine life [41], but those cases represent *M. aeruginosa* originating from a freshwater reservoir in a river system that are flushed into the coastal region instead of growth in a more saline environment. In the current study, however, the mostly unicellular *M. aeruginosa* strains were capable of growth at elevated salinity, which clearly shows the capacity to thrive at elevated salinity. 

Salttolerance in *M. aeruginosa* has been ascribed to genotypes harboring sucrose genes [42,43]. It has been postulated that recent horizontal gene transfer occurred when brackish water became more eutrophic [43] and that strains isolated from brackish water may possess genes against salt stress [42]. Strain MBC was isolated from brackish water and its salt tolerance may thus likely be a result of possession of sucrose genes. MTV was harvested from a fishpond in Trà Vinh, which had rather low salinity at the moment of sampling (0.5‰). However, this region was one of the most severely impacted ones [36] and the close vicinity to the CổChiên River that experienced salt intrusion up to 65 km inland enhances the possibility that this strain has also experienced more saline conditions in the fishpond in the past. The isolated strains have not been tested for presence of genes against salt stress, but they evidently were capable of coping with higher salinity as both strains were maintained in normal WC medium for months before the experiment was conducted. The gradual increase in salinity (4‰ per 7 days) may have favoured adaptation to higher salinities [44], which seems a more likely scenario in natural environments than a shockapproach of immediately transferring cells from the WC medium (0.5‰) to high salinities of 12‰ and 16‰. A replacement of salinity-sensitive cells by salinity-resistant mutants in *M. aeruginosa* was less likely, because with 10^−7^–10^−6^ mutants per generation [45], a sharp decline in biomass would have occurred when salinity was increased. 

**Figure A1:**
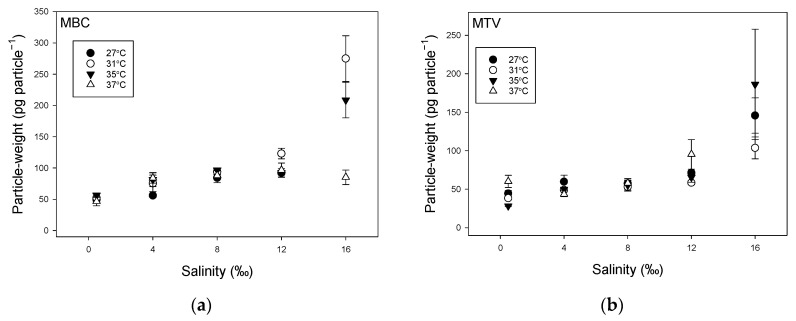
(**a**) Steady state particle weights (pg particle^−1^) of *Microcystis aeruginosa* strain MBC grown at different salinities (0.5–16‰) and four temperatures (27 °C–37 °C). Values are means of three consecutive days. Error bars indicate 1SD; (**b**) Steady state particle weights (pg particle^−1^) of *M. aeruginosa* strain MTV grown at different salinities (0.5–16‰) and four temperatures (27 °C–37 °C). Values are means of three consecutive days. Error bars indicate 1SD.

In strain MBC, culture dry-weight concentrations were higher at 12‰ than at 0.5‰ (see Figure 1a); 1.4, 1.7, 1.4 and 1.4 times at 27 °C, 31 °C, 35 °C, and 37 °C, respectively. Likewise, in strain MTV this was 1.9, 2.3, 1.6 and 1.4 times at 27 °C, 31 °C, 35 °C, and 37 °C, respectively. Adding sea salt also meant additional nutrients were added to the medium and as such could lead to a higher carrying capacity. Sea water contains on average 0.7 mg L^−1^ nitrate and 0.1 mg L^−1^ phosphate [46], which is relatively low compared to the WC medium used (14 mg N L^−1^ and 1.5 mg P L^−1^) and means that in 12‰ medium the added N was approximately 0.26 mg L^−1^ and P around 0.04 mg L^−1^. Hence, higher carrying capacity dry-weight at elevated salinity is most likely not a result of added nutrients. Since particle concentrations did not increase with salinity, individual particles became heavier at higher salinities (Figure A1), which may reflect storage of carbohydrates, such as sucrose, as internal osmotica [47].

This study yielded proof that the two isolated *M. aeruginosa* strains could grow and maintain high biomass in virtually all of the tested temperatures and salinity combinations. However, the MC concentrations and quota showed more pronounced differences between treatments. Particularly, high temperature drastically reduced MC concentrations. For instance, when averaged over all salinities, MC concentrations in strain MBC at 31 °C were on average 57% of the concentrations at 27 °C, while they were as low as 7.5% at 35 °C, and 1% at 37 °C. Such strong impact of temperature on MC concentration and cell quota is in agreement with previous findings for the same strains [19] and is corroborated by findings for other strains [18,48,49]. It has been postulated that lower MC cell quota at higher temperatures may be the result of binding of MCs to proteins [48], as higher temperatures may cause oxidative stress, which is mitigated by binding of MC to proteins [50]. 

The effect of salinity on MC concentrations and cell quota was less clear. MC concentrations remained either similar or increased with salinity rise from 0.5 to 8‰ (or 12‰), but in all treatments MC concentrations were lowest in cultures grown at 16‰. Similar results have been obtained for *M. aeruginosa* PCC 7806, that showed unaffected growth and MC quota of ~45 fg cell^−1^ until a salinity of 10‰, but a decline in both at higher salinities [25]. Another study, using the same strain, observed constant growth until a salinity of 8.4‰, while MC cell quota only declined at 16.9‰ [30]. However, in *M. aeruginosa* PCC 7820 growth and MC cell quota declined with increasing salinity [30], whereas no difference was found in MC per cell of *M. aeruginosa* strain Sj reared at 0 and 7.5‰ [43]. In general, it seems that lower MC concentrations and MC quota are found close to salt levels that reduced growth and thus created salt stress, as salinity stress may have a negative effect on the transcription of microcystin synthetase genes [51].

## 4. Conclusions

In conclusion, temporary increases in temperature and salinity in freshwater ecosystems do not seem detrimental to *M. aeruginosa* already present in the water bodies in southern Vietnam. *M. aeruginosa* could maintain high biomass up to relatively high salinities of 12‰ or even 16‰. Inasmuch as these higher salinities will occur during the warmer dry period, the warmer temperatures may lead to lower MC quota. The overall toxicity of a bloom in situ will be determined by the biomass of toxigenic strains and their toxin quota. Since MC quota may drop sharply in water temperatures of ≥35 °C, more blooms or higher biomass do not necessarily mean higher health risk. Nonetheless, the current occurrence of toxigenic *Microcystis* in southern Vietnam already presenta health risk [5] and with projected climate change this situation will likely worsen. Since climate change seems difficult to tackle, mitigating eutrophication that provides the fuel for the nuisance blooms seems a no-regret policy [52].

## 5. Materials and Methods

### 5.1. Sampling Locations and Microcystis Strains

**Figure 5:**
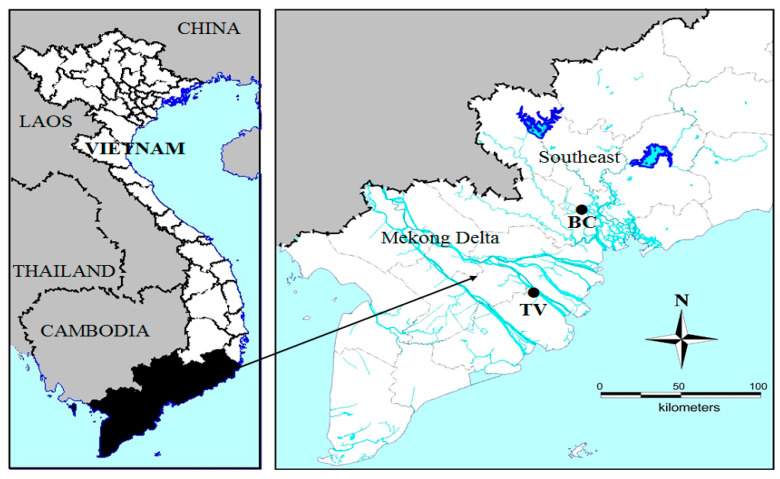
Location of the sampling sites where *Microcystis* strains MBC and MTV were collected in southern Vietnam. BC = fishpond in BinhChanh District (HoChiMinh City), TV = fishpond in Mekong Delta (TraVinh province).

Two strains of *Microcystis* were collected and isolated during bloom events, one was from a fishpond in BinhChanh district, HoChiMinh city (so called MBC), the other one was from a fishpond in TraVinh (so called MTV), a province located in the Mekong delta (see Figure 5). At each collecting site, temperature, salinity and pH were measured by a portable WTW 340i meter (WTW, Weilheim, Germany). Chlorophyll-*a* was measured with an AlgaeTorch (bbeMoldaenke GmbH, Schwentinental, Germany, see Appendix E for calibration). Samples from the fishponds were measured in a bucket after dilution of collected scum material with tap water to remain within the advised measuring range for the AlgaeTorch. Scum samples and water samples were transported to the laboratory for further analysis. Nutrients in the field water samples were analysedcolorimetrically with a spectrophotometer (Hach R/2010) using the following APHA (2005) [53] methods: nitrate 4500NO_3_^−^, ammonium 4500NH_4_^+^, total nitrogen (TN) Kjeldahl 4500N, phosphate and total phosphorus (TP) 4500P. The detection limits of the equipment for these water quality variables were 0.02 mg L^−1^ (nitrate), 0.04 mg L^−1^ (ammonium), 0.06 mg L^−1^ (TN Kjeldahl) and 0.05 mg L^−1^ for both TP and phosphate.

The analyses revealed that these two fishponds were highly eutrophic as indicated by high total nitrogen (TN), total phosphorus (TP) and high cyanobacteria chlorophyll-*a* concentrations (Table 1). The ponds were also characterised by high water temperatures and relatively high pH. The salinity of the surface water at the moment of sampling indicated that the isolated strain MBC could be referred to as a brackish strain while the isolated strain MTV was a freshwater strain (Table 1).

In the laboratory, single *Microcystis* colonies were picked out of the collected scum material by the micropipette-washing method [54]. These *Microcystis* strains were grown in small glass tubes with a few mL of modified WC medium [55] for several months at 25 °C, under a 14:10 h light/dark cycle at a light intensity of 70 µmol photon m^−2^ s^−1^. When strains reached a greenish appearance, they were transferred into 50 mL Erlenmeyer flasks and further cultivated.

### 5.2. Growth Experiment

**Figure 6:**
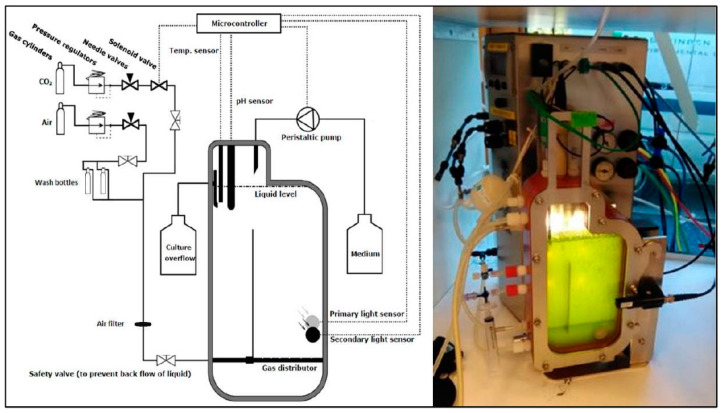
Schematic representation of the Algaemist flat-photobioreactors used in the experiment (**left**) and a picture of a reactor operational during the experiment (**right**).

The two *Microcystis* strains were first acclimatised to the experimental conditions (see below) for a week. Then they were inoculated in 300 mL Erlenmeyer flasks that contained 200 mL WC medium and closed with a cellulose plug. The flasks were put in the Sanyo Gallenkamp orbital incubators (MLR-351H, SANYO Electric Co., Ltd., Osaka, Japan) at 27 °C (low), 31 °C (medium), 35 °C (high) and 37 °C (extreme high temperature). After 4 days of culture, when the *Microcystis* strains were in exponential growth phase, they were inoculated in flat-panel airlift photobioreactors (Algaemist, Technical Development Studio, Wageningen University, Wageningen, The Netherlands; Figure 6) with the initial concentration of 52 ± 3 µg L^−1^ chlorophyll-*a*. The Algaemist has a 45 mm chamber with a volume of approximately 1250 mL. The reactor vessel is illuminated by a LEDpanel (BXRA W1200, Bridgelux, Fremont, CA, USA) in the control unit; light intensities can be set in the range 0–2500 µmol m^−2^ s^−1^.

Four Algaemists were used for strain MBC and four for strain MTV. For each strain, one system was run at low temperature (27 °C), one at medium (31 °C), one at high (35 °C) and one at extreme temperature (37 °C). Culture temperature levels were automatically maintained by thermal sensors, an internal heater and cooling from a Julabo FE500 water bath (Julabo Labortechnik GmbH, Seelbach, Germany) connected to the water jacket (internal length of 13 mm) at the front of the reactor. The pH was kept ˂9.0 by means of continuous pH measurements (probe QP150X/12 × 50/6X150, Prosense) and CO_2_ supply (grade 4.0, LindeGas). Moisturized compressed air (600 mL/min) was used to keep the culture in suspension; both gasses were passed through a WattmanPolyVent filter (PTFE, 0.2 µm) before entering the culture. The experiment started with normal WC medium (0.5‰) at four temperatures for both strains, which means eight systems were run. After one week, when cultures had reached steady state, samples were taken on three consecutive days. Thereafter, salinity was increased to 4‰ by adding sea salt to the medium (Jozo sea salt, Nouryon, Amsterdam, The Netherlands); cultures were allowed one week to reach steady state and again samples were taken on three consecutive days. This procedure was repeated for 8‰, 12‰ and 16‰ salinity. The reactors were operated in chemostat mode supplied with 0.361 (±0.017) liter WC medium per day, which equals a dilution rate of 0.289 (±0.011) d^−1^ (Appendix D, Table A4) and a light intensity of 100 µmol m^−2^ s^−1^ under 14:10 h light/dark cycle.

When the *Microcystis* reached steady state, 15 mL culture material was filtered over two separate glass-fibre filters (Whatman GF/C), of which one was stored at −20 °C for MC analysis (see Section 5.3). The other filter that had been weighed before filtration was put in an oven at 60 °C for 48 h and subsequently the dry-weight was measured on a 0.01 mg resolution balance (Sartorius R 160P, Göttingen, Germany). Additionally, subsamples of 2.5 mL were also taken to analyse the chlorophyll-*a* concentration and Photosystem-II (PSII) efficiency using a PHYTO-PAM phytoplankton analyser (HeinzWalz GmbH, Effeltrich, Germany) and particleconcentrations using a CASY counter (Casy TTC, Schärfe System, GmBh, Reutlingen, Germany). The particle concentration can be expressed as equivalent cell density, whichwas applied to calculate microcystin concentrations in *Microcystis* cells.

### 5.3. Microcystin (MC) Analysis 

The frozen filters stored at −20 °C were transferred to 8 mL glass tubes and dried for two hours in a freeze-drier (Alpha 1–2 LD, Martin Christ Gefriertrocknungsanlagen GmbH, Osterode am Harz, Germany). The filters were extracted three times at 60 °C in 2.5 mL of 75% methanol–25% Millipore water (*v/v*). The extracts were then dried in the Speedvac (Savant SPD121P, Thermo Scientific, Waltham, MA, USA) and subsequently reconstituted in 900 µL 100% methanol. The reconstituted samples were transferred to 2 mL Eppendorf vials with a cellulose-acetate filter (0.2 µm, Grace Davison. Discovery Sciences, Deerfield, IL, USA) and centrifuged for 5 min at 16,000× *g* (VWR Galaxy 16DH, VWR International, Buffalo Grove, IL, USA). Filtrates were then transferred to amber glass vials for LC-MS/MS analysis. 

Concentrations of eight MC variants (dm-7-MC-RR, MC-RR, MC-YR, dm-7-MC-LR, MC-LR, MC-LY, MC-LW and MC-LF) and nodularin (NOD) were determined by LC-MS/MS on an Agilent 1200 LC and an Agilent 6410A QQQ (Agilent Technologies, Santa Clara, CA, USA) as described in detail elsewhere [56]. Certified analytical standards (DHI LAB Products, Hørsholm, Denmark) were used to quantify MCs.

MC concentrations were expressed per unit volume (µg L^−1^), but also based on biomass/cell density indicators, as MC per particle, MC per unit chlorophyll-*a* and as mass fraction of dry-weight.

## Figures and Tables

**Table 1:** Water temperature (°C), salinity (‰), pH, total nitrogen (TN), total phosphorus (TP), dissolved nutrients and chlorophyll-*a* (CHLa) concentrations in two fishponds from which *Microcystis aeruginosa* strains MBC and MTV were isolated.

Strain	Temp. (°C)	Salinity (‰)	pH	TN (mg L^−1^)	TP (mg L^−1^)	N-NH_4_ (mg L^−1^)	N-NO_3_ (mg L^−1^)	P-PO_4_ (mg L^−1^)	CHLa (µg L^−1^)
MBC	37.4	7.5	9.77	9.00	0.33	0.67	<0.01	<0.02	1520
MTV	33.1	0.5	9.58	19.5	1.75	1.14	0.28	0.02	4352

**Figure A2:**
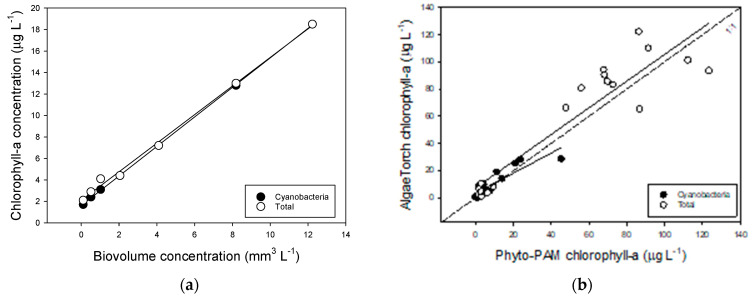
(**a**) Relationship between the biovolume concentration of a laboratory culture of the cyanobacterium *Anabaena cylindrica* PCC7122 and the cyanobacterial and total chlorophyll-*a* concentrations determined with the AlgaeTorch. (**b**) Relationships between the cyanobacterialand total chlorophyll-*a* concentrations determined with the Phyto-PAM and with the AlgaeTorch.

## Data Availability

Not applicable.

## Appendix A

**Table A1:** Results of linear regressions of the dry-weight (DW) of two *M. aeruginosa* strains grown at different salinities (0.5–12‰), and at four temperatures, including the *r*^2^ of the fits.

Strain	Temp (°C)	DW = DW_0_ + a × Sal	*r* ^2^
MBC	27	287.9 + 11.91 × Sal	0.9375
	31	281.6 + 18.57 × Sal	0.9844
	35	291.3 + 12.77 × Sal	0.6967
	37	325.2 + 11.09 × Sal	0.9807
MTV	27	228.4 + 26.05 × Sal	0.8876
	31	251.3 + 24.25 × Sal	0.8612
	35	290.7 + 16.13 × Sal	0.9141
	37	255.4 + 17.81 × Sal	0.9487

**Table A2:** Parallel lines analysis for the course of biomass indicators (particle concentration, chlorophyll-*a* concentration and dry-weight concentration) of the two *M. aeruginosa* strains MBC and MTV grown at 4 incubation temperatures at salinities of 0.5‰, 4‰, 8‰ and 12‰.

Indicator		*M. aeruginosa* MBC	*M. aeruginosa* MTV
Particles	slope	*F*_3,8_ = 0.3903; *p* = 0.7634	*F*_3,8_ = 1.7188; *p* = 0.2400
	intercept	*F*_3,11_ = 1.0647; *p* = 0.4034	*F*_3,11_ = 2.0534; *p* = 0.1648
Chlorophyll-*a*	slope	*F*_3,8_ = 2.6897; *p* = 0.1170	*F*_3,8_ = 0.3372; *p* = 0.7992
	intercept	*F*_3,11_ = 0.7597; *p* = 0.5398	*F*_3,11_ = 1.6620; *p* = 0.2322
Dry-weight	slope	*F*_3,8_ = 1.0432; *p* = 0.4245	*F*_3,8_ = 0.8387; *p* = 0.5097
	intercept	*F*_3,11_ = 1.4221; *p* = 0.2887	*F*_3,11_ = 0.4524; *p* = 0.7208

## Appendix B

**Table A3:** Share (%) of different MC variants detected in *M. aeruginosa* strain MBC grown at different salinities (0.5–12‰) and at four temperatures (27, 31, 35, 37 °C).

*Microcystis aeruginosa* MBC						
Temp (°C)	Sal. ‰	dmMC-RR	MC-RR	MC-YR	dmMC-LR	MC-LR
27	0.5	4.2	31.4	12.9	2.8	48.8
27	4	2.6	24.6	12.1	2.7	58.0
27	8	1.2	21.9	11.6	1.3	64.0
27	12	1.8	23.4	11.5	1.0	62.3
27	16	wash out *	---*	---*	---*	---*
31	0.5	4.2	32.3	14.0	2.4	47.1
31	4	1.9	27.7	13.8	1.3	55.2
31	8	1.5	27.4	13.6	1.0	56.6
31	12	1.9	28.6	10.9	0.7	57.9
31	16	2.4	29.0	13.1	1.4	54.2
35	0.5	15.5	43.2	8.6	<LOQ	32.7
35	4	7.8	30.9	11.2	<LOQ	50.1
35	8	5.7	28.3	11.8	<LOQ	54.2
35	12	6.2	31.9	10.4	<LOQ	51.5
35	16	8.8	33.6	10.5	<LOQ	47.1
37	0.5	<LOQ	<LOQ	11.6	<LOQ	88.4
37	4	<LOQ	68.3	6.2	<LOQ	25.5
37	8	<LOQ	67.1	6.1	<LOQ	26.8
37	12	<LOQ	61.6	5.5	<LOQ	32.9
37	16	<LOQ	49.3	7.3	<LOQ	43.4

* indicates a wash out of the culture at given condition.

## Appendix D

**Table A4:** WC medium supplied to the AlgaeMist systemsoperated at different temperatures. Vessels were weighed daily (1 L WC medium = 1 kg) and the dilution rate was determined by dividing the added volume by the vessels operating volume (1.25 L).

WC Medium Supplied (L per Day)							
MBC				MTV			
27 °C	31 °C	35 °C	37 °C	27 °C	31 °C	35 °C	37 °C
0.340	0.366	0.349	0.391	0.362	0.347	0.332	0.387
0.354	0.368	0.351	0.389	0.353	0.366	0.338	0.388
0.369	0.366	0.351	0.386	0.362	0.345	0.347	0.387
0.367	0.363	0.348	0.385	0.347	0.354	0.352	0.390
0.370	0.361	0.344	0.403	0.364	0.356	0.347	0.413
0.366	0.363	0.356	0.401	0.356	0.358	0.348	0.374
0.365	0.364	0.355	0.398	0.339	0.357	0.353	0.370
0.369	0.363	0.353	0.397	0.358	0.352	0.349	0.366
0.360	0.362	0.360	0.425	0.364	0.352	0.358	0.366
0.358	0.357	0.352	0.328	0.350	0.351	0.356	0.365
0.362	0.361	0.355	0.395	0.323	0.353	0.348	0.370
0.363	0.358	0.352	0.361	0.364	0.348	0.345	0.364
0.358	0.346	0.338	0.359	0.360	0.334	0.351	0.352
0.364	0.384	0.381		0.376	0.378	0.340	0.394
0.366	0.354	0.350			0.347	0.354	0.366
0.357	0.351	0.350			0.346		0.362
Averages added (L)/dilution rate (d^−1^)				Averages added (L)/dilution rate (d^−1^)			
0.362 L	0.362 L	0.353 L	0.386 L	0.356 L	0.353 L	0.348 L	0.377 L
0.289 d^−1^	0.289 d^−1^	0.282 d^−1^	0.309 d^−1^	0.284 d^−1^	0.282 d^−1^	0.278 d^−1^	0.301 d^−1^

## Appendix E

The AlgaeTorch was tested in the laboratory using a culture of *Anabaena cylindrica* PCC7122 of which the biovolume concentration was determined using the CASY counter. Different portions of culture material were diluted in 2 L medium and the cyanobacterial- and total chlorophyll-*a* concentrations were measured with the AlgaeTorch. This yielded: cyanobacterial chlorophyll-*a* = 1.6178 + 1.3745 × Biovolume (*r*^2^ = 0.999); total chlorophyll-*a* = 2.0946 + 1.3306 × Biovolume (*r*^2^ = 0.997) (Figure A2a). Subsequently, the AlgaeTorch (AT) was taken into the field where cyanobacterial—and total chlorophyll-*a* concentration were measured while a sample of the water was also analyzed on a PHYTO-PAM (PP) phytoplankton analyzer (PHYTO-ED, Heinz Walz GmbH, Effeltrich, Germany) that had been calibrated against the Dutch standard [57], which is a hot ethanol extraction based on (Moed and Hallegraeff 1978). This yielded acceptable relations for cyanobacterial chlorophyll-*a*: AT = 3.3067 + 0.7399 × PP (*r*^2^ = 0.784), and for total chlorophyll-*a*: AT = 7.2522 + 0.9816 × PP (*r*^2^ = 0.855) (Figure A2b).
